# Supplementary material for: Clinical Remission in Severe Asthma: A Comparative Analysis of Patients with and Without Biologics from the Swiss Severe Asthma Registry
Source: Biomedicines. 2025 Dec 12;13(12):3074. doi: 10.3390/biomedicines13123074 (PMC12730426; doi:10.3390/biomedicines13123074)
Supplement: Supplementary file 1 [file biomedicines-13-03074-s001.zip › biomedicines-3976365-supplementary.pdf]

biomedicines

MDPI

Supplementary Material

Table S1. Imputed and non-imputed variables used for the regression models including the extent of missingness per variable.

Variable	N missing	N Total	% Missing	Data type	Imputation
BEC	200	394	50.76	Numerical	No
Biologic duration	96	394	24.37	Numerical	No
Asthma in Relatives	55	394	13.96	Categorical	No
BEC prior Biologics	45	394	11.42	Numerical	No
FEV1 (% predicted)	27	394	6.85	Numeric	Yes
FEV 1 (l)	25	394	6.35	Numeric	Yes
Beclomethasone equivalent	24	394	6.09	Numeric	Yes
Number of Exacerbations	16	394	4.06	Numeric	Yes
ACT Score	12	394	3.05	Numeric	Yes
Childhood onset	9	394	2.28	Categorical	Yes
Lowest FEV 1 last 2 years (l)	8	394	2.03	Numeric	Yes
Lowest FEV 1 last 2 years (% predicted)	7	394	1.78	Numeric	Yes
GERD	5	394	1.27	Categorical	Yes
Nasal Polys	4	394	1.02	Categorical	Yes
COPD	4	394	1.02	Categorical	Yes
Depression	4	394	1.02	Categorical	Yes
Allergies	3	394	0.76	Categorical	Yes
BMI	2	394	0.51	Numeric	Yes
OCS	1	394	0.25	Categorical	Yes
Age	0	394	0	Other	Yes
Sex	0	394	0	Categorical	Yes
Biologic treatment	0	394	0	Other	Yes
Biologic past	0	394	0	Other	Yes

Abbreviations: BEC: Blood Eosinophil Count; BMI: Body Mass Index; COPD: Chronic Obstructive Pulmonary Disease; GERD: Gastroesophageal Reflux Disease; ICS: Inhaled Corticosteroids; OCS: Oral Corticosteroids. Childhood onset: before the age of 12. Asthma in first degree relatives (mother, father, siblings, own children).

Table S2. Full table of patient characteristics in both groups.

	Biologic-Naïve (n= 96)	Biologic-Treated (n= 298)	p-value
Female, n (%)	51 (53.1%)	131 (44%)	0.15
Age, Mean (SD)	52.92 (16.02)	55.63 (15.67)	0.13
BMI, Mean (SD)	26.86 (5.73)	27.85 (5.71)	0.06
Underweight, n (%)	5 (5.2%)	8 (2.7%)	0.11
Normal weight, n (%)	24 (25%)	110 (37.2%)	
Overweight, n (%)	37 (38.5%)	106 (35.8%)	
Obese, n (%)	30 (31.2%)	72 (24.3%)	
**Type of Asthma**			
Allergic, n (%)	33 (34.4)	147 (49.3)	0.02
Non-Allergic, n (%)	43 (44.8)	96 (32.2)	
Mixed, n (%)	20 (20.8)	55 (18.5)	
**Asthma and family history**			
Childhood-Onset[a], n (%)	69 (23.7)	24 (25.5)	0.82
Duration of Asthma, geom.mean (GSD)[b]	5.84 (2.7)	11.09 (2.36)	< 0.001

	**Biologic-Naïve (n= 96)**	**Biologic-Treated (n= 298)**	**p-value**
**Vaccination Status**			
Pneumococcus, n (%)	23 (24.5)	73 (24.7)	0.84
Influenza, n (%)	44 (46.3)	179 (60.5)	< 0.01
**Smoking Status**			
Lifelong Non-Smoker, n (%)	52 (54.2)	159 (53.4)	
Former-Smoker, n (%)	29 (30.2)	118 (39.6)	0.02
Current Smoker, n (%)	15 (15.6)	21 (7)	
**Disease Burden**			
Patients that miss Work/School, n (%)	17 (17.9)	47 (15.9)	0.77
ACT Score, mean (SD)	17.23 (4.45)	19.98 (4.68)	< 0.001
No Symptoms, n (%)	4 (4.2)	69 (23.2)	< 0.001
**Exacerbations in the previous year**			
Number of Exacerbations, geom. mean (GSD) b	1.09 (2.02)	0.49 (1.80)	< 0.001
No Exacerbation	38 (39.6)	187 (62.8%)	
≤ 2 Exacerbations per year	32 (33.3)	81 (27.2%)	< 0.001
> 2 Exacerbations per year	26 (27.01)	30 (10.1%)	
**Medication**			
High-dose ICS, n (%)	68 (78.2%)	253 (89.4%)	0.01
Beclomethasone Equivalent (µg/day) , Mean (SD)	1417 (786)	1345 (873)	0.33
OCS, n (%)	17 (17.9)	52 (17.4)	1
Prednisolone Equivalent (mg/day), Mean (SD)	14.4 (12.8)	11.6 (15)	0.03
**Pulmonary Function**			
FEV1 (l) , Mean (SD)	2.23 (0.76)	2.43 (0.87)	0.10
FEV1 (% predicted) , Mean (SD)	72.33 (19.55)	77.59 (20.38)	< 0.01
FVC (l) , Mean (SD)	3.33 (0.93)	3.58 (0.99)	0.07
FVC (% predicted), Mean (SD)	86.33 (15.92)	90.92 (16.05)	0.01
Lowest FEV1 in the last 2 years (l) , Mean (SD)	1.98 (0.68)	2.07 (0.84)	0.73
Lowest FEV1 in the last 2 years (% predicted) , Mean (SD)	65.51 (20.10)	65.33 (20.58)	0.91
BHR, n (%)	31 (33)	88 (29.5)	0.61
**Laboratory findings**			
Current BEC (cells/µL), geom.mean (GSD)b	203.34 ± 5.23	29.57 ± 13.93	< 0.001
**Remission Criteria**			
ACT Score ≥ 20, n (%)	31 (33.7%)	190 (65.5%)	< 0.001
No OCS, n (%)	79 (82.3)	246 (82.6)	1
FEV1 > 80% predicted, n (%)	26 (27.1)	132 (44.3)	< 0.01
No Exacerbations, n (%)	35 (36.5)	178 (59.7)	< 0.001
Fulfilling all 4 remission criteria, n (%) [c]	6 (6.2)	72 (24.2)	< 0.001
Fulfilling 3 remission criteria, n (%) [d]	15 (15.6)	136 (45.7)	< 0.001
**Co-Morbidities**			
Allergies, n (%)	45 (48.4)	158 (53)	0.51
Sinusitis, n (%)	33 (35.5%)	143 (48.1%)	0.04
Nasal Polyps, n (%)	14 (15.1%)	117 (39.4%)	< 0.001
GERD, n (%)	19 (20.4%)	79 (26.7%)	0.28
Depression, n (%)	13 (14%)	31 (10.4%)	0.45
COPD, n (%)	12 (12.9%)	30 (10.1%)	0.56
EGPA, n (%)	1 (1.1%)	17 (5.7%)	0.08
ABPA, n (%)	5 (5.4%)	9 (3%)	0.45
Bronchiectasis of unknown origin, n (%)	6 (6.5%)	24 (8.1%)	0.77
> 2 LRTIs/ year, n (%)	14 (15.1%)	44 (14.8%)	1

	Biologic-Naïve (n= 96)	Biologic-Treated (n= 298)	p-value

Abbreviations: ABPA: Allergic bronchopulmonary aspergillosis; ACT: Asthma Control Test; BEC: Blood Eosinophil Count; BHR: Bronchial Hyperresponsiveness; BMI: Body Mass Index; COPD: Chronic Obstructive Pulmonary Disease; EGPA: Eosinophilic Granulomatosis with Polyangiitis; GERD: Gastroesophageal Reflux Disease; ICS: Inhaled Corticosteroids; LRTI: Lower Respiratory Tract Infection; OCS: Oral Corticosteroids.

[a] Childhood onset: before the age of 12. BMI categories: Underweight BMI < 17.5 kg/m2 ; Normal Weight BMI 17.5-25 kg/m2; Overweight BMI 15-30 kg/m2 ; Obese > 30 kg/m2

[b] geometric mean and geometric standard deviation (GSD) was used, if the variable was not normally distributed

[c] Remission with 4 criteria: ACT controlled, no exacerbations, no OCS, FEV1 > 80 % predicted

[d] Remission with 3 criteria: ACT controlled, no exacerbations, no OCS,

Table S3. Predictors for Asthma Control (ACT score ≥ 20): Logistic Regression Results with Robust Standard Errors, imputed vs. complete case.

	Imputed Data Set				Complete Case			
	OR	95 % CI lower	95 % CI upper	p- value	OR	95 % CI lower	95 % CI upper	p- value
Allergies	0.456	0.195	1.066	0.07	0.515	0.219	1.211	0.128
Age	0.996	0.971	1.022	0.783	1.001	0.974	1.028	0.951
Asthma in Relatives	0.562	0.275	1.148	0.114	0.522	0.24	1.138	0.102
BMI	0.977	0.916	1.043	0.484	0.977	0.913	1.045	0.489
Biologic Treatment	3.959	1.483	10.564	**0.006**	3.516	1.256	9.838	**0.017**
Childhood Onset	1.02	0.443	2.348	0.963	1.252	0.494	3.173	0.636
BEC	1	1	1	0.952	1	0.999	1.001	0.94
Female Sex	0.85	0.41	1.763	0.662	1.16	0.533	2.523	0.709
High Dose ICS	1.198	0.326	4.4	0.786	1.122	0.287	4.388	0.868
COPD	0.124	0.015	1.044	0.055	0.145	0.016	1.305	0.085
Depression	0.317	0.059	1.705	0.181	0.574	0.112	2.934	0.504
GERD	1.021	0.449	2.322	0.961	1.133	0.48	2.672	0.776
Nasal Polyps	1.59	0.747	3.387	0.229	1.573	0.69	3.585	0.281
Lowest FEV1 (%predicted)	1.006	0.99	1.022	0.457	1.013	0.996	1.031	0.124
Biologic Switcher	0.386	0.17	0.879	**0.023**	0.397	0.162	0.975	**0.044**

Abbreviations: BEC: Blood Eosinophil Count; BMI: Body Mass Index; COPD: Chronic Obstructive Pulmonary Disease; GERD: Gastroesophageal Reflux Disease; ICS: Inhaled Corticosteroids; OCS: Oral Corticosteroids. Childhood onset: before the age of 12. Asthma in first degree relatives (mother, father, siblings, own children). **Model Specifications:** GLM; Family Binominal, Link Function: Logit; Estimation Maximum Likelihood with robust standard errors. **Outcome:** Asthma Control (ACT score ≥ 20).

Table S4. Predictors for No OCS use: Logistic Regression Results with Robust Standard Errors, imputed vs. complete case.

	Imputed Data Set				Complete Case			
	OR	95 % CI lower	95 % CI upper	p- value	OR	95 % CI lower	95 % CI upper	p- value
Allergies	0.712	0.273	1.859	0.488	0.803	0.301	2.143	0.661
Age	0.987	0.95	1.025	0.489	0.991	0.954	1.029	0.629
Asthma in Relatives	1.057	0.431	2.595	0.903	1.628	0.567	4.675	0.365
BMI	1.144	1.035	1.264	**0.009**	1.151	1.031	1.285	**0.012**
Biologic Treatment	6.268	1.983	19.813	**0.002**	11.518	2.796	47.454	**0.001**
Childhood Onset	1.597	0.366	6.96	0.533	1.08	0.24	4.849	0.92
BEC	1.001	0.999	1.003	0.204	1.001	0.999	1.003	0.31

Female Sex	1.096	0.386	3.116	0.863	0.834	0.288	2.417	0.738
High Dose ICS	0.877	0.142	5.435	0.888	0.544	0.057	5.179	0.596
COPD	0.343	0.077	1.525	0.16	0.22	0.044	1.093	0.064
Depression	0.816	0.139	4.799	0.822	0.577	0.082	4.071	0.581
GERD	0.587	0.174	1.985	0.392	0.424	0.138	1.302	0.134
Nasal Polyps	0.924	0.352	2.423	0.872	0.792	0.284	2.209	0.656
Lowest FEV1 (%predicted)	1.024	1	1.049	0.055	1.019	0.992	1.046	0.177
Biologic Switcher	0.203	0.062	0.672	**0.009**	0.148	0.038	0.568	**0.005**

Abbreviations: BEC: Blood Eosinophil Count; BMI: Body Mass Index; COPD: Chronic Obstructive Pulmonary Disease; GERD: Gastroesophageal Reflux Disease; ICS: Inhaled Corticosteroids; OCS: Oral Corticosteroids. Childhood onset: before the age of 12. Asthma in first degree relatives (mother, father, siblings, own children). **Model Specifications:** GLM; Family Binominal, Link Function: Logit; Estimation Maximum Likelihood with robust standard errors. **Outcome:** No OCS use.

Table S5. Predictors for FEV 1 > 80 % predicted: Logistic Regression Results with Robust Standard Errors, imputed vs. complete case.

	Imputed Data Set				Complete Case			
	OR	95 % CI lower	95 % CI upper	p- value	OR	95 % CI lower	95 % CI upper	p- value
Allergies	0.507	0.162	1.586	0.243	0.523	0.149	1.832	0.311
Age	1.016	0.986	1.046	0.291	1.006	0.974	1.038	0.719
Asthma in Relatives	1.131	0.378	3.379	0.825	0.918	0.257	3.272	0.895
BMI	0.99	0.913	1.084	0.916	0.968	0.888	1.056	0.464
Biologic Treatment	4.423	1.392	14.056	**0.011**	5.503	1.439	21.052	**0.013**
Childhood Onset	1.03	0.338	3.147	0.955	0.812	0.294	2.246	0.689
BEC	1	0.999	1	0.074	1	0.999	1	0.2
Female Sex	2.465	0.872	6.964	0.088	2.831	0.95	8.435	0.062
High Dose ICS	0.372	0.072	1.910	0.236	0.388	0.087	1.742	0.217
COPD	0.216	0.043	1.072	0.061	0.42	0.092	1.922	0.264
Depression	0.368	0.076	1.777	0.213	0.209	0.042	1.047	0.057
GERD	0.181	0.049	0.666	**0.010**	0.282	0.068	1.164	0.08
Nasal Polyps	1.306	0.402	4.243	0.656	0.916	0.276	3.044	0.886
Lowest FEV1 (%predicted)	1.139	1.091	1.191	< **0.001**	1.124	1.075	1.174	< **0.001**
Biologic Switcher	0.443	0.139	1.403	0.166	0.393	0.119	1.293	0.124

Abbreviations: BEC: Blood Eosinophil Count; BMI: Body Mass Index; COPD: Chronic Obstructive Pulmonary Disease; GERD: Gastroesophageal Reflux Disease; ICS: Inhaled Corticosteroids; OCS: Oral Corticosteroids. Childhood onset: before the age of 12. Asthma in first degree relatives (mother, father, siblings, own children). **Model Specifications:** GLM; Family Binominal, Link Function: Logit; Estimation Maximum Likelihood with robust standard errors. **Outcome:** FEV1 > 80 % predicted.

Table S6. Predictors for Absence of Exacerbations: Logistic Regression Results with Robust Standard Errors, imputed vs. complete case.

	Imputed Data Set				Complete Case			
	OR	95 % CI lower	95 % CI upper	p- value	OR	95 % CI lower	95 % CI upper	p- value
Allergies	0.827	0.389	1.759	0.623	0.62	0.278	1.383	0.243
Age	1.00	0.975	1.026	0.968	1.009	0.981	1.037	0.531
Asthma in Relatives	0.613	0.300	1.252	0.179	0.648	0.301	1.393	0.266

BMI	0.996	0.933	1.064	0.927	1.006	0.939	1.078	0.87
Biologic Treatment	5.107	2.007	12.996	**0.001**	5.124	1.961	13.389	**0.001**
Childhood Onset	0.685	0.296	1.583	0.377	0.814	0.323	2.049	0.662
BEC	1	1	1.001	0.290	1	1	1.001	0.705
Female Sex	0.856	0.421	1.742	0.668	0.819	0.38	1.766	0.61
High Dose ICS	2.112	0.559	7.982	0.270	1.561	0.359	6.788	0.552
COPD	0.427	0.111	1.643	0.216	0.228	0.042	1.223	0.084
Depression	3.404	0.962	12.04	0.057	3.855	0.922	16.11	0.064
GERD	1.492	0.688	3.233	0.309	1.497	0.676	3.316	0.32
Nasal Polyps	1.03	0.498	2.145	0.927	0.824	0.373	1.82	0.633
Lowest FEV1 (%predicted)	1.004	0.988	1.021	0.576	1.005	0.988	1.022	0.55
Biologic Switcher	0.627	0.281	1.396	0.253	0.569	0.228	1.419	0.226

Abbreviations: BEC: Blood Eosinophil Count; BMI: Body Mass Index; COPD: Chronic Obstructive Pulmonary Disease; GERD: Gastroesophageal Reflux Disease; ICS: Inhaled Corticosteroids; OCS: Oral Corticosteroids. Childhood onset: before the age of 12. Asthma in first degree relatives (mother, father, siblings, own children). **Model Specifications:** GLM; Family Binominal, Link Function: Logit; Estimation Maximum Likelihood with robust standard errors. **Outcome:** Absence of Exacerbations.
